# CircPRKD3/miR-6783-3p responds to mechanical force to facilitate the osteogenesis of stretched periodontal ligament stem cells

**DOI:** 10.1186/s13018-024-04727-7

**Published:** 2024-04-22

**Authors:** Jiani Liu, Rui Liu, Hong Wang, Zijie Zhang, Jixiao Wang, Fulan Wei

**Affiliations:** Department of Orthodontics, School and Hospital of Stomatology, Cheeloo College of Medicine, Shandong University & Shandong Key Laboratory of Oral Tissue Regeneration & Shandong Engineering Research Center of Dental Materials and Oral Tissue Regeneration & Shandong Provincial Clinical Research Center for Oral Diseases, No. 44-1 Wenhua Road West, Jinan, Shandong 250012 China

**Keywords:** CircPRKD3, miR-6783-3p, ceRNAs, PDLSCs, Mechanical force

## Abstract

**Background:**

The mechanotransduction mechanisms by which cells regulate tissue remodeling are not fully deciphered. Circular RNAs (circRNAs) are crucial to various physiological processes, including cell cycle, differentiation, and polarization. However, the effects of mechanical force on circRNAs and the role of circRNAs in the mechanobiology of differentiation and remodeling in stretched periodontal ligament stem cells (PDLSCs) remain unclear. This article aims to explore the osteogenic function of mechanically sensitive circular RNA protein kinase D3 (circPRKD3) and elucidate its underlying mechanotransduction mechanism.

**Materials and methods:**

PDLSCs were elongated with 8% stretch at 0.5 Hz for 24 h using the Flexcell® FX-6000™ Tension System. CircPRKD3 was knockdown or overexpressed with lentiviral constructs or plasmids. The downstream molecules of circPRKD3 were predicted by bioinformatics analysis. The osteogenic effect of related molecules was evaluated by quantitative real-time PCR (qRT‐PCR) and western blot.

**Results:**

Mechanical force enhanced the osteogenesis of PDLSCs and increased the expression of circPRKD3. Knockdown of circPRKD3 hindered PDLSCs from osteogenesis under mechanical force, while overexpression of circPRKD3 promoted the early osteogenesis process of PDLSCs. With bioinformatics analysis and multiple software predictions, we identified hsa-miR-6783-3p could act as the sponge of circPRKD3 to indirectly regulate osteogenic differentiation of mechanically stimulated PDLSCs.

**Conclusions:**

Our results first suggested that both circPRKD3 and hsa-miR-6783-3p could enhance osteogenesis of stretched PDLSCs. Furthermore, hsa-miR-6783-3p could sponge circPRKD3 to indirectly regulate RUNX2 during the periodontal tissue remodeling process in orthodontic treatment.

**Supplementary Information:**

The online version contains supplementary material available at 10.1186/s13018-024-04727-7.

## Introduction

External mechanical force can mediate the reconstruction of alveolar bone tissue. In this process, periodontal ligament stem cells (PDLSCs) play a critical role in converting mechanical force into biological signals for bone remodeling [1]. Stretch-induced PDLSCs can differentiate into osteoblasts, secreting a series of cytokines to regulate the differentiation and maturation of osteoclasts, which in turn controls the balance between osteoclastogenesis and osteogenesis, changing the direction of bone reconstruction ultimately [2]. Therefore, studying and elucidating the osteogenic differentiation mechanism of PDLSCs under mechanical force is the basis for further exploring the periodontal tissue remodeling process.

To date, noncoding RNAs (ncRNAs) have attracted widespread attention due to their up-to-75% proportion of human genome [3]. Common ncRNAs mainly included microRNAs (miRNAs), small nucleolar RNAs (snoRNAs), ribosomal RNAs (rRNAs), transfer RNAs (tRFs), long noncoding RNAs (lncRNAs), and circular RNAs (circRNAs) [4]. They played different roles in gene regulation [5], cell development [6], disease outcome [7, 8], and metabolism control [9]. Our previous reports clarified the inhibitory effects of miR-21 [10] and lncRNA SNHG8- EZH2 axis on mechanically stimulated osteogenesis of PDLSCs based on the high-throughput sequencing results [11]. Subsequently, we focused on circRNAs with particular ring structures, which enabled them to escape RNase degradation and maintain high stability [12]. Although the circRNA expression profiles under mechanical force were mainly enriched in transcription, translation, energy metabolism, cellular signaling and communication [13], their specific regulatory modes remain to be further explored. Incremental studies demonstrated that circRNAs could act as competing endogenous RNAs (ceRNAs) by sponging miRNAs to change their target genes expression in the osteogenesis of mesenchymal stem cells (MSCs) [14, 15]. We demonstrated formerly that miR-21 could regulate the osteogenesis of PDLSCs under mechanical force through ACVR2B [16]. Based on the proof that circRNAs could act as “sponges” of miRNAs, we predicted circRNAs related to miR-21 and selected seven functional circRNAs for validation. And circular RNA protein kinase D3 (circPRKD3) among the prediction results changed most significantly in PDLSCs under mechanical load.

CircPRKD3 was a 943nt circRNA, whose function has not yet been reported. However, PRKD3, as the parental gene of circPRKD3, was demonstrated to be dysregulated during tumorigenesis, functioning as a potential oncogene [17]. Further studies showed PRKD3 altered macrophage polarization and the production of transforming growth factor beta in cirrhotic liver tissues [18]. In addition, PRKD3 was involved in the pro-inflammatory process regulated by MMP1/13, playing an important role in cartilage matrix degradation [19] and the differentiation of multinucleated mature osteoclasts [20]. Given that PRKD3 was related to the bone repair process, we inferred that circPRKD3, a transcription product of PRKD3, might be involved in the osteogenic development of MSCs. Nonetheless, the function and mechanotransduction mechanism of circPRKD3 involved in the osteogenesis of mechanically stimulated PDLSCs need further study.

In this research, we shed light on the role of circPRKD3 and miR-6783-3p in bone formation by mechanical stretch, and demonstrated that circPRKD3 and miR-6783-3p could interact as a “sponge” to indirectly regulate periodontal osteogenesis under mechanical force. These findings provide novel insights into the mechanotransduction mechanism of circRNAs and their impacts on stem cell pluripotency, which hold significant implications for mechanically induced periodontal tissue regeneration, as well as craniomaxillofacial and embryonic development.

## Methods

### Cell culture and identification

The study was approved by the Medical Ethical Committee at the School of Stomatology, Shandong University (No. GR201710). Third molars without periodontitis, caries, or pulpitis were collected from 20 patients aged 13–18, with their parents’ written informed consent. As previously described, PDLSCs were obtained and cultivated [21]. The expression of stem cell surface markers was assessed to identify the stem cell characteristics of PDLSCs by flow cytometry. According to prior procedures, PDLSCs were cultivated in specific osteogenic and adipogenic mediums to examine the multi-directional differentiation capacity [22]. Briefly, the osteogenesis ability of PDLSCs was assessed by alkaline phosphatase (ALP) activity and extracellular matrix calcification, detected through ALP staining and Alizarin red staining. The adipogenic differentiation capacity of PDLSCs was estimated by Oil Red O staining for identifying lipid-rich adipocytes.

### Application of mechanical force

The Flexcell® FX-6000™ Tension System (Flexcell International Corporation, Burlington, NC, USA) was utilized to apply mechanical stimuli. Briefly, PDLSCs were seeded on Flexcell Amino silicone-bottomed plates at 2.0 × 10^5^ cells/well with α-MEM, containing 10% fetal bovine serum. When the cells reached 80% confluence, the above plates were placed into the tension system, and an 8% stretch at 0.5 Hz was applied for 24 h. Meanwhile, plates without stress were set as the negative control group.

### Cell transfection

CircPRKD3 knockdown was performed by lentiviral transfection. Lentiviral constructs (Genechem, Shanghai, China) were designed according to different regions of the circPRKD3 sequence (sh-circPRKD3 group). The same lentiviral vector containing aspecific RNA oligonucleotide was grouped into the negative control (sh-NC group). PDLSCs were transfected with the above lentiviral vectors at an optimized multiplicity of infection (MOI) of 20.

CircPRKD3 overexpression was conducted by plasmid transfection. The augmented circPRKD3 was spliced into pLC5-ciR (Geneseed, Guangzhou, China) with EcoRI and BamHI (pLC5-circPRKD3 group). The same plasmid vector containing non-specific RNA oligonucleotide was also used as the negative control (pLC5-ciR-NC group). Plasmid transfection was achieved by the transfection-promoting reagents Zlip2000 (Zoman Biotechnology, Beijing, China) and lip2000 (Invitrogen).

miR-6783-3p mimics was synthesized as specified to achieve the overexpression of miR-6783-3p (General Biosystems, Anhui, China). The transfection concentration with control mimics (NC-mimics group) and miR-6783-3p mimics (mimics-miR-6783-3p group) was 20 µM following the manufacturer’s protocol.

### Quantitative real-time PCR (qRT‐PCR)

SYBR® Premix Ex Taq™ (Takara) was used to conduct the qRT-PCR reaction in accordance with the manufacturer’s instructions. Briefly, the treated PDLSCs were lysed with Trizol (Takara). Subsequently, chloroform, isopropanol, and absolute ethanol were added in sequence to obtain RNA precipitation. Then, cDNA generated by 1 µg RNA was obtained following the instructions of the reverse transcription kit (Accurate Biology, AG). Table 1 provided a list of the primer sequences, including GAPDH, ALP, RUNX2, circPRKD3, U6, has-miR-208a-5p, has-miR-214-3p, has-miR-761, has-miR-3619-5p, has-miR-4761-5p, has-miR-5003-3p, has-miR-6516-5p, has-miR-6783-3p, has-miR-7855-5p, has-miR-8075. GAPDH was employed as a standardizing control for mRNAs, whereas U6 was utilized as a normalizing control for miRNAs. The experiments were conducted using three biological replicates, each with 2–3 technical replicates. Relative quantification was calculated following the comparative 2^−ΔΔCt^ method.

**Table 1:** Primers used for qRT-PCR

Primer name	Sense primers (5’-3’)	Antisense primers (3’-5’)
**GAPDH**	GCACCGTCAAGGCTGAGAAC	TGGTGAAGACGCCAGTGGA
**ALP**	GGACCATTCCCACGTCTTCA	CAGGCCCATTGCCATACA
**RUNX2**	TCCACACCATTAGGGACCATC	TGCTAATGCTTCGTGTTTCCA
**circPRKD3**	CCATTGAAGCCCAGGAAC	GCTGATGCTTTCTGACATATAG
**U6**	GGAACGATACAGAGAAGATTAGC	TGGAACGCTTCACGAATTTGCG
**miR-208a-5p**	GAGCTTTTGGCCCGGGTTATAC	
**miR-214-3p**	AGCAGGCACAGACAGGCAGT	
**miR-761**	CAGCAGGGTGAAACTGACACA	
**miR-3619-5p**	CAGGCAGGTTGGTGCAGCAAA	
**miR-4761-5p**	TTACGTGTGCATGCCTGACC	
**miR-5003-3p**	TACTTTTCTAGGTTGTTGGGG	
**miR-6516-5p**	GGCAGTAACAGGTGTGAGCA	
**miR-6783-3p**	TTCCTGGGCTTCTCCTCTGTAG	
**miR-7855-5p**	TGAGGACCCCAAGCTCGGAAA	
**miR-8075**	TTGCTGATGGCAGATGTCGGGT	

### Western blot

Following two rinses with pre-chilled PBS, PDLSCs were lysed using the mixed reagent composed of RIPA solution, 1% PMSF and 1% phosphatase inhibitor. The protein samples were then transferred onto polyvinylidene difluoride (PVDF) membranes after being sorted on 10% sodium dodecyl sulfate-polyacrylamide gel electrophoresis according to their size and electrical properties. Blocking PVDF membranes with 5% milk for two hours to reduce nonspecific binding. Afterward, membranes were coated with selected antibodies at 4 °C for 12-16 h. The antibodies involved in this paper mainly include GAPDH (1:10000, Abcam), ALP (1:1000, HuaBio), and RUNX2 (1:1000, CST). The next day, secondary antibodies (1:10000, Proteintech) were utilized to incubate with primary antibodies bound to membranes for 1 h at room temperature. Subsequently, protein bands were imaged with chemiluminescence reagents in a gel imaging system (Amersham Imager 600, General Electric Company). The protein expression was quantified by Image J analysis. The original images of western blots supporting the conclusions of this article are included within the additional file 1.

### Dual-luciferase reporter assay

293T cells were transfected as above described. Relative luciferase activity was measured according to the instructions of dual-luciferase reporter assay kits (Vazyme). Briefly, the transfected 293T cells were treated with cell lysis buffer. Afterwards the collected supernatant was added into luciferase substrate to detect the activity of firefly luciferase. Finally, adding renilla substrate into the above solution to detect the activity of renilla luciferase as a parameter for correcting transfection efficiency.

### Statistical analysis

Data were presented quantitatively as mean ± standard deviation (SD) of three independent experiments. Difference between two groups was analyzed with Student’s t test, while for three or more groups, one-way analysis of variance (ANOVA) tests were performed. *P* < 0.05 was conceived as statistically significant.

## Results

### Identification and biological characteristics of PDLSCs

**Fig. 1:**
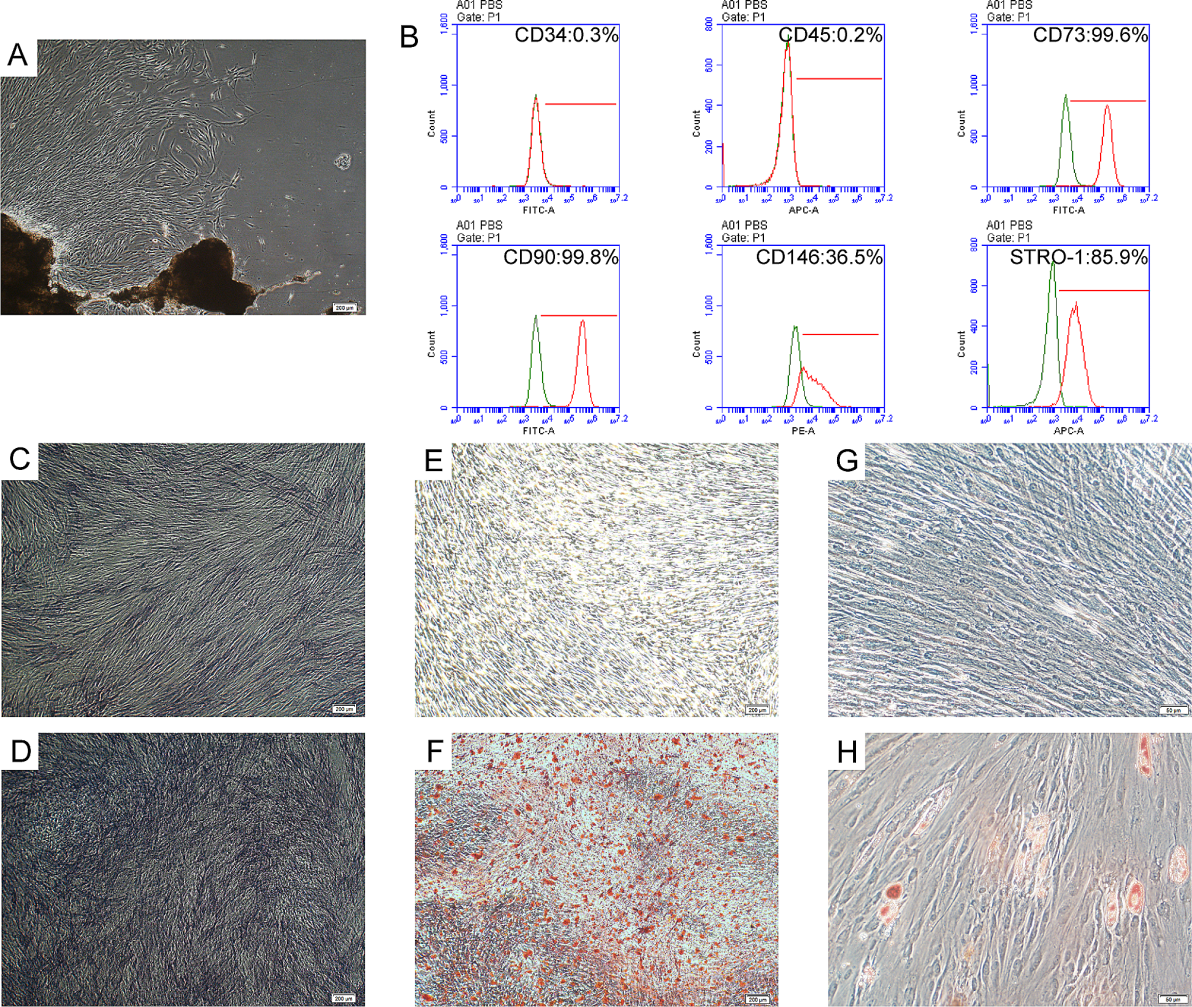
Identification and biological characteristics of PDLSCs. (**A**) Morphology of PDLSCs (P0) from periodontal tissue blocks (scale bar 200 μm). (**B**) Identification of MSCs surface markers in PDLSCs by flow cytometry. ALP staining of PDLSCs cultured for 7 days without (**C**) or with (**D**) osteogenic induction (scale bar 200 μm). Alizarin red staining of PDLSCs cultured for 28 days without (**E**) or with (**F**) osteogenic induction (scale bar 200 μm). Oil red O staining of PDLSCs cultured for 21 days without (**G**) or with (**H**) adipogenic induction (scale bar 50 μm)

Long spindle-shaped PDLSCs were obtained from isolated periodontal tissue blocks (Fig. 1A). Flow cytometry showed the positive expression of mesenchymal stem cell surface markers in PDLSCs, including CD73, CD90, CD146, and STRO-1, meanwhile the expression of hematopoietic stem cell marker CD34 and leukocyte marker CD45 were negative (Fig. 1B). Elevated ALP activity was detected by ALP staining (Fig. 1C, D), while increased extracellular matrix calcification was discovered by Alizarin Red staining, which demonstrated that PDLSCs possessed osteogenic differentiation capacity (Fig. 1E, F). The lipid droplets displayed by Oil Red O staining proved that PDLSCs had the ability of adipogenic differentiation (Fig. 1G, H). The above results fully proved the multi-directional differentiation potential of PDLSCs. And all the results indicated that PDLSCs isolated from periodontal ligament tissue met the basic standard of mesenchymal stem cells.

### CircPRKD3 was upregulated during the osteogenic development of mechanically stimulated PDLSCs

**Fig. 2:**
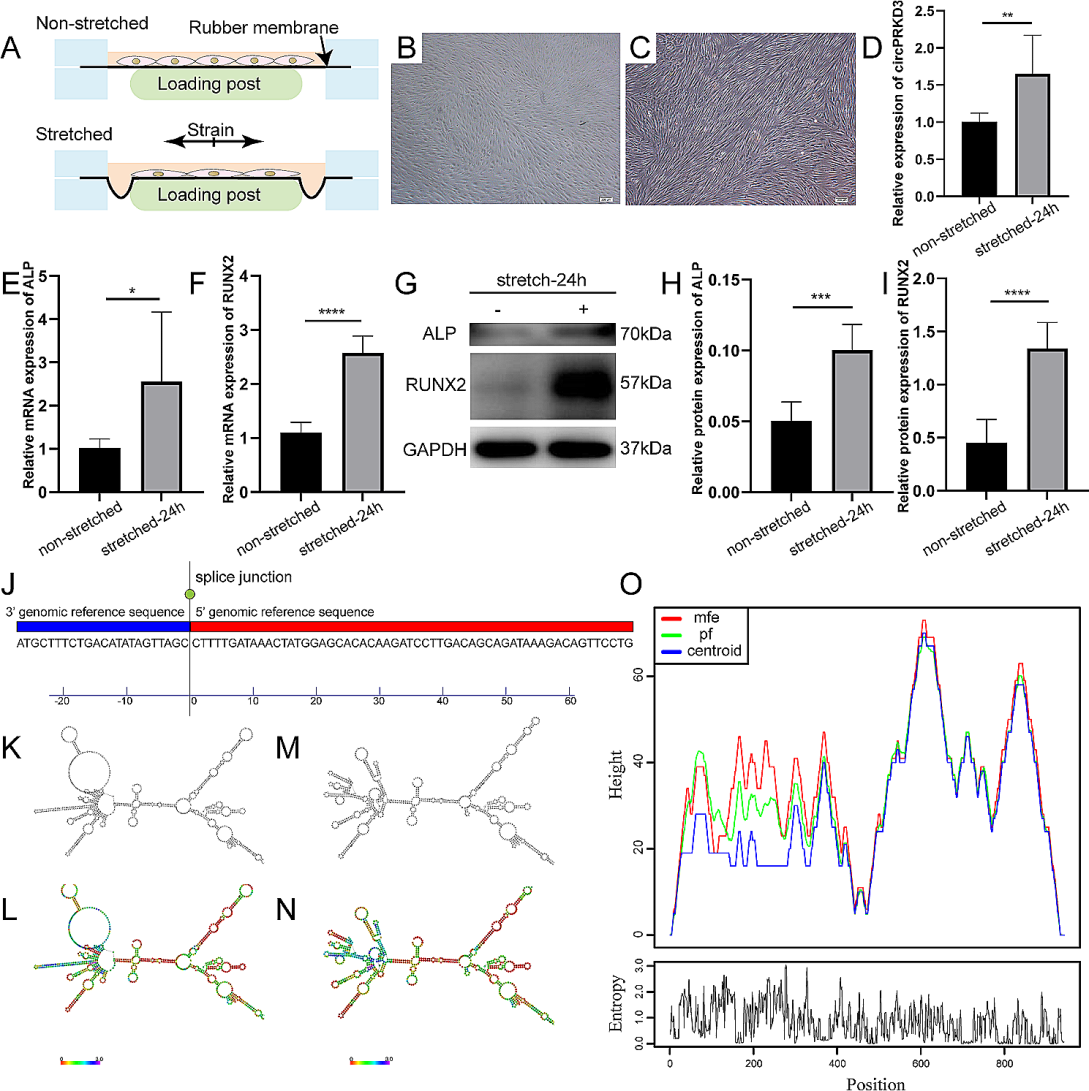
CircPRKD3 was upregulated during osteogenic development of mechanically stimulated PDLSCs. (**A**) Schematic diagram of mechanically stimulated PDLSCs in vitro. Morphology of PDLSCs without (**B**) or with (**C**) stretch inducement for 24 h (scale bar 200 μm). (**D**) Relative gene expression level of circPRKD3 in non-stretched and stretched-24 h groups. (**E**-**F**) The mRNA expression level of ALP and RUNX2 in non-stretched and stretched-24 h groups. (**G**-**I**) The protein level of ALP and RUNX2 and the quantitative data analyzed by image J in non-stretched and stretched-24 h groups. Full-length blots were presented in Supplementary Fig. 1 of additional file 1. (**J**) The genetic sequence of circPRKD3 on circBase. (**K**) Centroid plain structure drawing of circPRKD3. (**L**) Centroid structure drawing encoding positional entropy of circPRKD3. (**M**) MFE plain structure drawing of circPRKD3. (**N**) MFE structure drawing encoding positional entropy of circPRKD3. (**O**) Peak diagram of circPRKD3 with minimum free energy. Data are presented as mean ± SD of three independent experiments. (*p < 0.05; **p < 0.01; ***p < 0.001; ****p < 0.0001)

The mechanically stimulated PDLSCs in vitro was schematically shown in Fig. 2A. Compared with PDLSCs arranged randomly without stretch inducement (Fig. 2B), PDLSCs under mechanical force were arranged along the force direction with stretched morphology (Fig. 2C). As shown in Fig. 2E-I, the application of mechanical force enhanced the osteogenesis potential of PDLSCs. And the result of Fig. 2D demonstrated that mechanical stimuli increased the expression of circPRKD3, which suggested the high possibility that circPRKD3 was involved in the osteogenesis process of stretch-induced PDLSCs. We subsequently searched for the splice junction of circPRKD3 on circBase (Fig. 2J) and the structure of circPRKD3 on RNAfold web server to learn more about the properties of circPRKD3. Interestingly, circPRKD3 with minimum free energy presented two predicted structures (Fig. 2K-O), which indicated the diversity of RNA secondary structure of circPRKD3.

### Regulation of circPRKD3 affected osteogenesis of mechanically stimulated PDLSCs

**Fig. 3:**
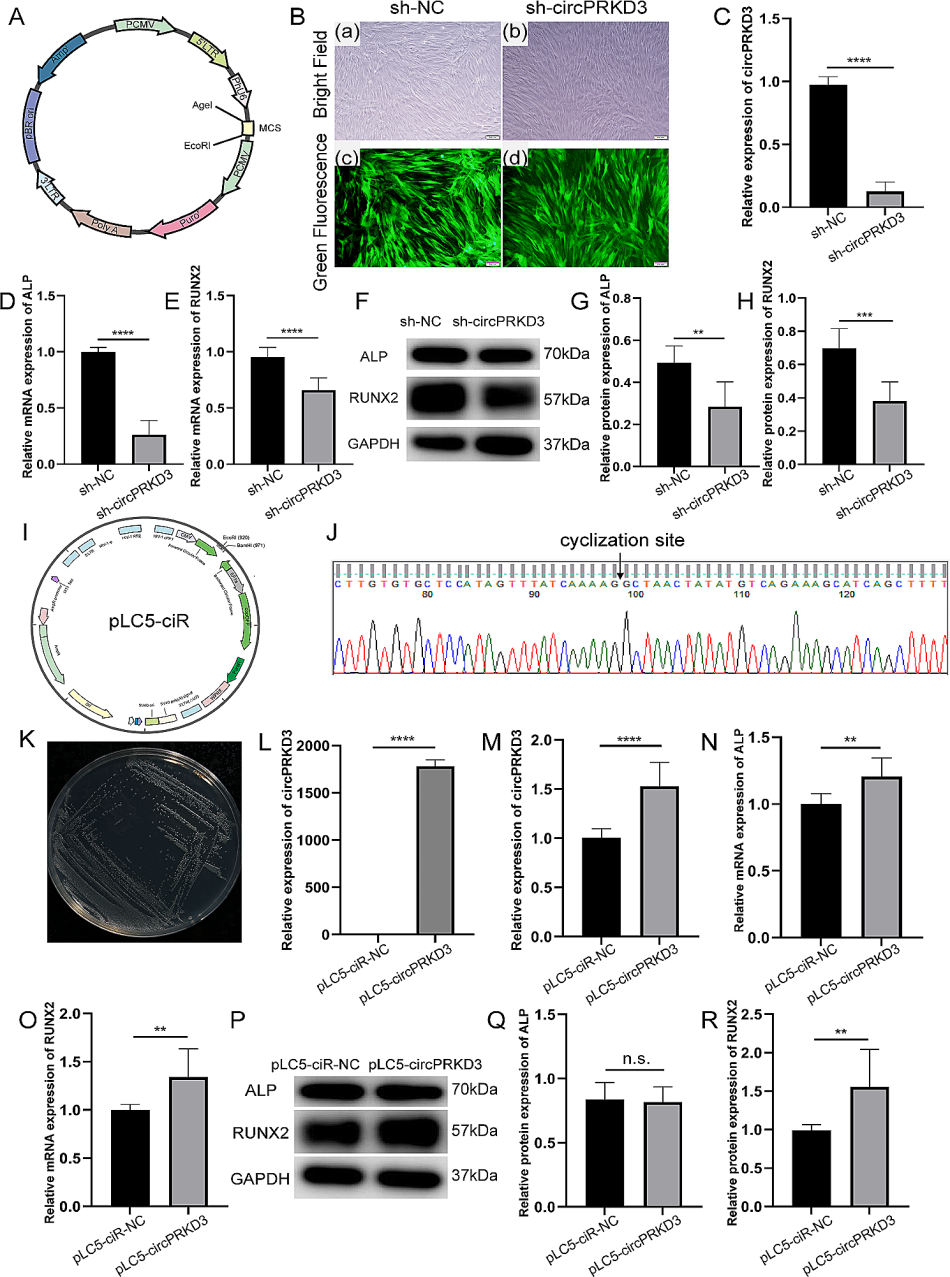
Regulation of circPRKD3 affected osteogenesis of mechanically stimulated PDLSCs. (**A**) Schematic diagram of virus construction. Immunofluorescence staining (**B**) and qRT-PCR verification (**C**) of transfection efficiency in sh-NC and sh-circPRKD3 groups. (**D**-**E**) The mRNA expression level of ALP and RUNX2 in mechanically stimulated sh-NC and sh-circPRKD3 groups after transfection for 24 h. (**F**-**H**) The protein level of ALP and RUNX2 and the quantitative data analyzed by image J in mechanically stimulated sh-NC groups and sh-circPRKD3 groups after transfection for 24 h. Full-length blots were presented in Supplementary Fig. 2 of additional file 1. (**I**) Schematic diagram of plasmids construction. (**J**) The head-to‐tail splicing of circPRKD3 as a qRT‐PCR product was verified by Sanger sequencing. (**K**) Pure E. coli containing circPRKD3-overexpressed plasmids were filtered out by ampicillin. Verification of transfection efficiency in 293T (**L**) and PDLSCs (**M**) was assayed by qRT‐PCR. (**N**-**O**) The mRNA expression level of ALP and RUNX2 in mechanically stimulated pLC5‐ciR-NC groups and pLC5‐circPRKD3 groups after transfection for 24 h. (**P**-**R**) The protein level of ALP and RUNX2 and the quantitative data analyzed by image J in mechanically stimulated pLC5‐ciR-NC groups and pLC5‐circPRKD3 groups after transfection for 24 h. Full-length blots were presented in Supplementary Fig. 3 of additional file 1. Data are presented as mean ± SD of three independent experiments. (n.s., no significance; **p < 0.01; ****p < 0.0001)

To further explore the function of circPRKD3 in the osteogenic process of mechanically stimulated PDLSCs, sequence target 57219-1 with the best downregulation effect was used to design and synthesize lentiviral vectors (Fig. 3A). The fluorescence imaging (Fig. 3B) and the result of qRT-PCR analysis (Fig. 3C) demonstrated effective knockdown of circPRKD3 with viral transfection.

To further examine whether down-regulated circPRKD3 could lessen the osteogenic differentiation potential of mechanically stimulated PDLSCs, sh-NC groups and sh-circPRKD3 groups were stimulated with the mechanical force for 24 h. The results revealed that suppressed circPRKD3 diminished the expression of ALP and RUNX2 at transcription (Fig. 3D, E) and translation levels (Fig. 3F-H), suggesting that downregulation of circPRKD3 inhibited stretch-induced osteogenesis of PDLSCs.

Based on the above results of circPRKD3 knockdown, we further specified its function by overexpressing circPRKD3. The full-length sequence of hsa_circ_0000992 (circPRKD3) was amplified and ligated into pLC5-ciR with EcoRI and BamHI (Fig. 3I). The synthetic sequence had no stray peaks or overlapping bands (Fig. 3J), suggesting the insertion of the genetic fingerprint of circPRKD3 into pLC5-ciR. Related plasmids were stored in glycerol bacteria, and pure E. coli containing circPRKD3-overexpressed plasmids were filtered out by ampicillin (Fig. 3K). After verification of plasmids extraction from E. coli by DNA purity, the overexpression efficiency of circPRKD3 was examined in 293T and PDLSCs (Fig. 3L, M). However, the transfection efficiency of PDLSCs was much lower than that of 293T, which might be due to different reactivity to transfection reagents between primary cells and cell lines.

Subsequently, pLC5-ciR-NC groups and pLC5-circPRKD3 groups were stimulated with the mechanical force for 24 h to further explore the osteogenic role of circPRKD3. The results showed that circPRKD3 overexpression reinforced RUNX2 expression at transcriptional and translation levels (Fig. 3O, P, R), while the expression of ALP was only upregulated at mRNA level (Fig. 3N, P, Q). The aforementioned findings demonstrated the initial pro-osteogenic function of circPRKD3 in PDLSCs induced by stretch.

### Screening and validation of circPRKD3 downstream targets

**Fig. 4:**
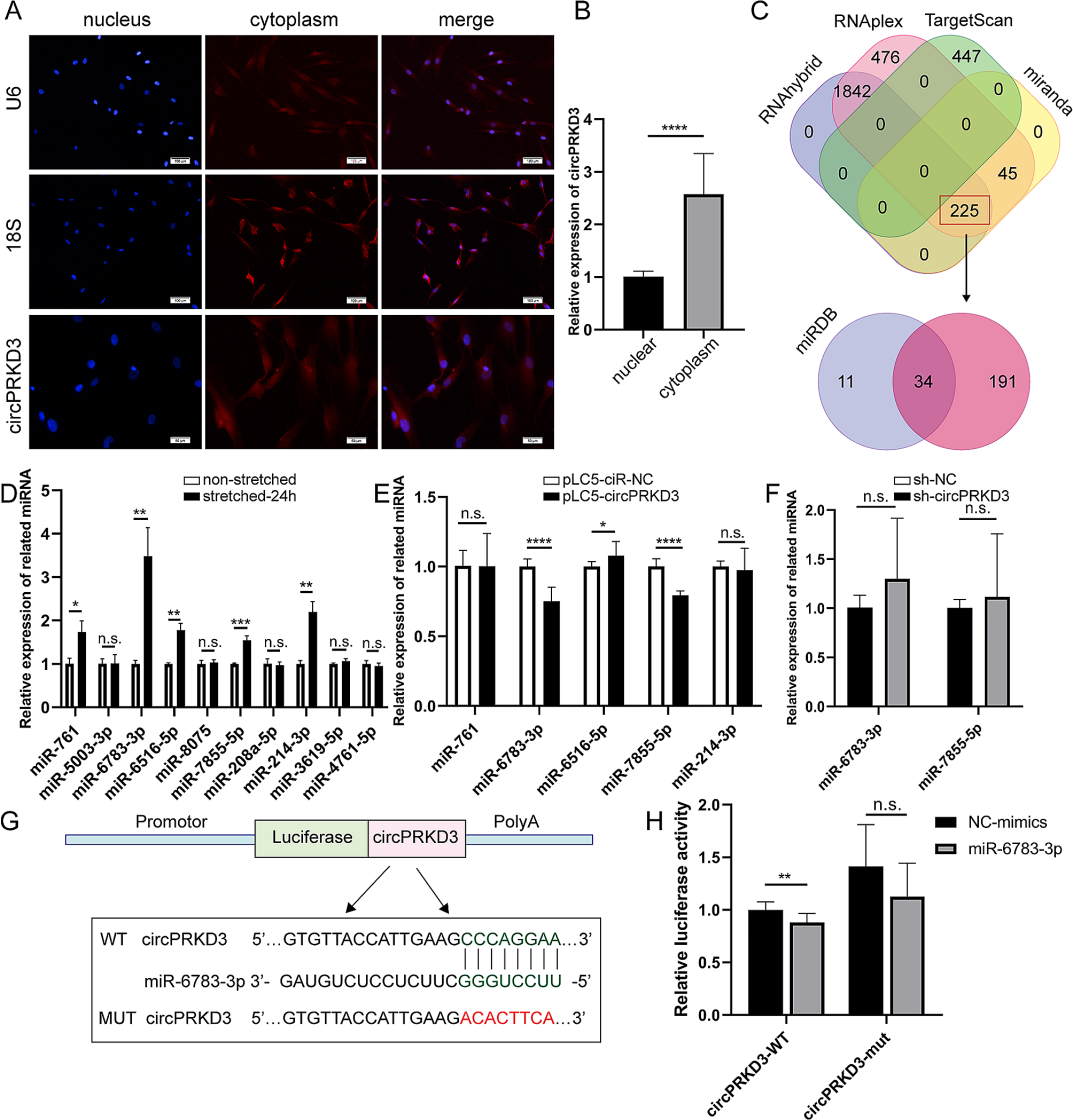
Screening and validation of circPRKD3 downstream targets. Immunofluorescence staining (**A**) and quantitative analysis (**B**) of circPRKD3 distribution in nucleus and cytoplasm. (**C**) Prediction of miRNAs combined with circPRKD3 through different algorithms. (**D**) Force-sensitive miRNAs were sought among the 10 predicted miRNAs above by qRT-PCR analysis. (**E**) CircPRKD3 overexpression-associated miRNAs among force-sensitive miRNAs were evaluated by qRT‐PCR in mechanically stimulated pLC5‐ciR-NC groups and pLC5‐circPRKD3 groups after transfection for 24 h. (**F**) CircPRKD3 knockdown-associated miRNAs among force-sensitive miRNAs were assayed by qRT‐PCR in mechanically stimulated sh-NC and sh-circPRKD3 groups after transfection for 24 h. (**G**) Schematic diagram of luciferase assay and related sequence information. (**H**) Relative luciferase activity was measured in circPRKD3-WT groups and circPRKD3-mut groups with or without miR-6783-3p mimics. Data are presented as mean ± SD of three independent experiments. (n.s., no significance; *p < 0.05; **p < 0.01; ***p < 0.001; ****p < 0.0001)

The nuclear/cytoplasmic distribution of circPRKD3 was explored subsequently to further investigate the mechanism of circPRKD3 function. Although fluorescent probe staining images showed that the distribution of circPRKD3 was throughout the nucleus and cytoplasm (Fig. 4A), qRT-PCR analysis verified a higher cytoplasmic proportion of circPRKD3 in PDLSCs through nucleocytoplasmic separation experiments (Fig. 4B). Considering miRNAs could combine with related circRNAs in the cytoplasm as a “sponge” and mediate their ability to regulate transcription process, we then focused on four algorithms, RNAhybrid, RNAplex, TargetScan, and Miranda, to predict miRNAs that might interact with circPRKD3. 225 related miRNAs were selected, and the miRDB database further narrowed the screening range to 34 miRNAs (Fig. 4C). Ultimately, 10 miRNAs were randomly selected based on the number of predicted binding sites and their functions. Finally, 5 mechanosensitive candidates, including miR-761, miR-6783-3p, miR-6516-5p, miR-7855 -5p, and miR-214-3p, were found to be upregulated in response to mechanical force (Fig. 4D), which was consistent with the upward trend of circPRKD3 induced by stretch.

We next evaluated miRNAs regulated by circPRKD3. The results identified that the expression of miR-6783-3p and miR-7855-5p was decreased with circPRKD3 overexpression (Fig. 4E). Nonetheless, both miR-6783-3p and miR-7855-5p showed insignificant change with circPRKD3 knockdown (Fig. 4F). Since miR-6783-3p altered most at the transcriptional level, possible binding sites between miR-6783-3p and circPRKD3 were summarized via miRDB database (Fig. 4G). Afterward, luciferase assay was carried out, and results exhibited that miR-6783-3p mimics reduced the luciferase activity of the circPRKD3-WT reporter, while that of the mutant reporter showed no statistically significant change (Fig. 4H), suggesting that circPRKD3 and miR-6783-3p could combine with each other.

### Interaction between miRNA-6783-3p and circPRKD3 regulated osteogenesis of mechanically stimulated PDLSCs

**Fig. 5:**
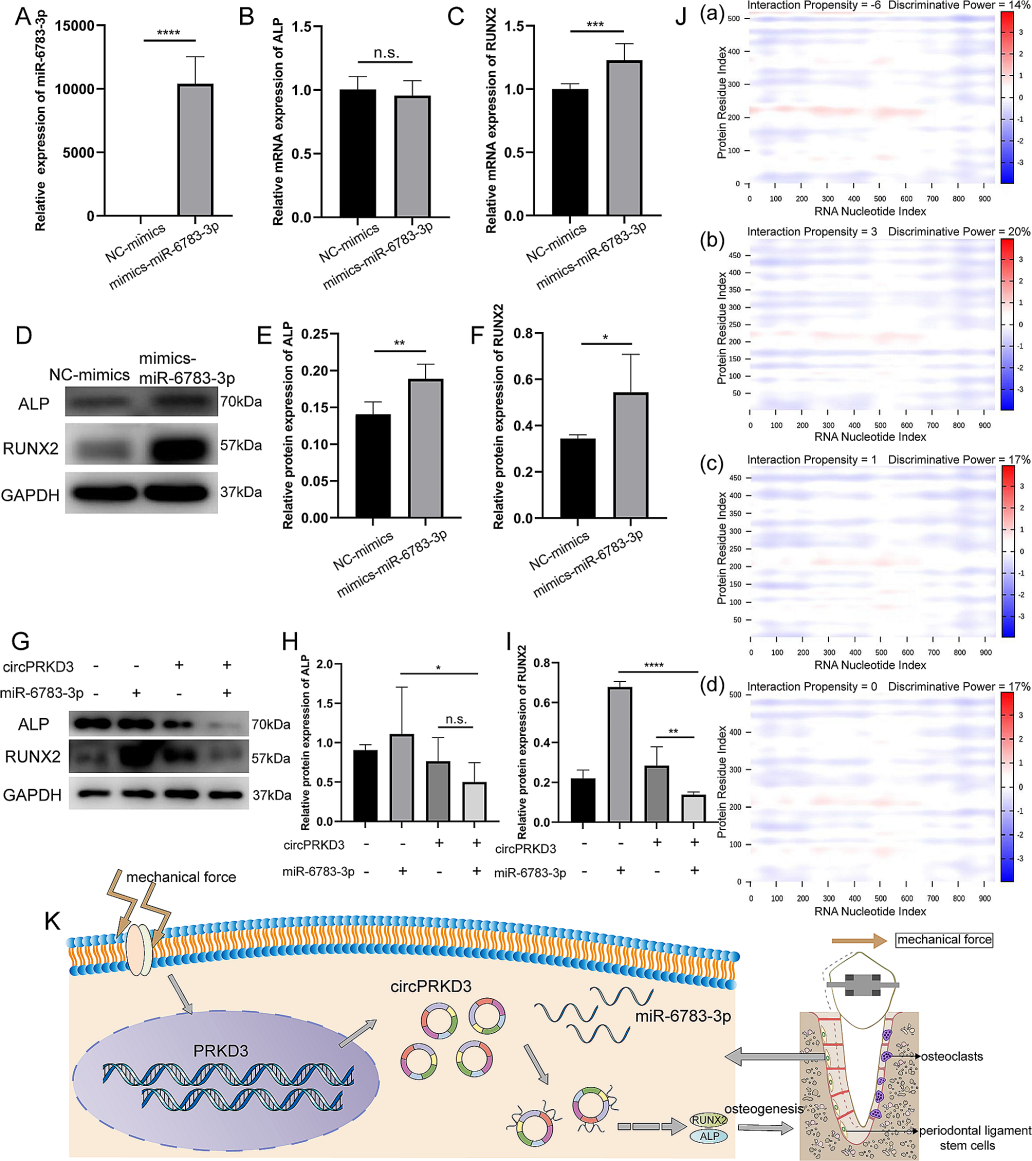
Interaction between miRNA-6783-3p and circPRKD3 regulated osteogenesis of mechanically stimulated PDLSCs. (**A**) Validation of miR-6783-3p overexpression efficiency by qRT-PCR analysis in PDLSCs. (**B**-**C**) The mRNA expression level of ALP and RUNX2 in mechanically stimulated NC-mimics groups and mimics-miR-6783-3p groups after transfection for 24 h. (**D**-**F**) The protein level of ALP and RUNX2 and the quantitative data analyzed by image J in mechanically stimulated NC-mimics groups and mimics-miR-6783-3p groups after transfection for 24 h. Full-length blots were presented in Supplementary Fig. 4 of additional file 1. (**G**-**I**) The protein level of ALP and RUNX2 and the quantitative data analyzed by image J after co-transfection of pLC5‐circPRKD3 plasmids and mimics-miR-6783-3p for 24 h under mechanical force. Full-length blots were presented in Supplementary Fig. 5 of additional file 1. (**J**) Predicted sites where circPRKD3 binds to different RUNX2 protein isoforms sequences on the catRAPID website. (a) Binding sites prediction on RUNX2 isoform a. (b) Binding sites prediction on RUNX2 isoform b. (c) Binding sites prediction on RUNX2 isoform d. (d) Binding sites prediction on RUNX2 isoform e. (**K**) The working model that miR-6783-3p acts as a sponge of circPRKD3 to indirectly regulate the osteogenesis of PDLSCs under mechanical force. Data are presented as mean ± SD of three independent experiments. (n.s., no significance; *p < 0.05; **p < 0.01; ***p < 0.001; ****p < 0.0001)

Although circPRKD3 and miR-6783-3p could target each other, the specific function of miR-6783-3p and whether miR-6783-3p could act as the sponge of circPRKD3 to participate in the osteogenesis of mechanically stimulated PDLSCs was unknown. Therefore, miR-6783-3p mimics was synthesized and confirmed to imitate the high-level expression of endogenous cellular miR-6783-3p and enhance its regulatory function (Fig. 5A). PDLSCs transfected with miR-6783-3p mimics were mechanically stimulated for 24 h to further analyze its function. The following results discovered that miRNA-6783-3p mimics increased the transcriptional expression of RUNX2 (Fig. 5C). However, the change of ALP expression level was not noticeable (Fig. 5B). Nevertheless, the expression of these two corresponding osteogenic markers were both multiplied at protein translation level (Fig. 5D-F). The above results confirmed that both miR-6783-3p and circPRKD3 exerted pro-osteogenic effects in PDLSCs under mechanical load.

The “sponge” relationship between miR-6783-3p and circPRKD3 was further verified next. Compared with groups where miR-6783-3p/circPRKD3 was transfected alone, the expression level of osteogenic protein decreased in the co-transfection group (Fig. 5G-I). This indicated that miR-6783-3p could adsorb circPRKD3 to regulate osteogenesis of PDLSCs induced by stretch. Since RUNX2 was more sensitive to the regulation of circPRKD3 and miR-6783-3p, we tried to detect whether the above two molecules could bind to its protein sequence and participate in osteogenesis directly. According to the prediction results of catRAPID (Fig. 5J) and TargetScan (Additional file 2), the above assumption was overturned. In conclusion, circPRKD3 and miR-6783-3p indirectly regulated the osteogenesis of mechanically stimulated PDLSCs by sponging each other (Fig. 5K).

## Discussion

PDLSCs could convert external mechanical stimuli into osteogenic biological signals, ultimately mediating periodontal tissue remodeling [23]. The osteogenesis under mechanical force was related to various factors, including soluble cytokines [24], signaling pathways [25], calcium-regulating hormones [26], immune responses [27], and inflammatory environment [28]. With the advancement of bioinformatics technology, more and more ncRNAs were verified to be involved in bone remodeling under mechanical force [29]. Recent evidence showed that diverse and conserved circRNAs were involved in biological development and disease progression, especially in different tissue regeneration [30–33]. However, how these mechanically sensitive circRNAs respond to mechanical stimuli and participate in the mechanotransduction of PDLSCs’ osteogenic differentiation induced by stretch has rarely been reported. Here, we identified circPRKD3 as a novel circular RNA sensitive to mechanical force, and revealed its mechanism by which circPRKD3 and miR-6783-3p could act as a “sponge” to indirectly regulate periodontal osteogenesis under mechanical force.

CircRNAs were produced by nonlinear back-splicing or gene rearrangement of precursor transcripts of parental genes [34]. Although the functional research of circPRKD3 has not been reported yet, the effects of circPRKD3 could be inferred by analyzing the function of its parental gene, PRKD3. PRKD3 belonged to the serine/threonine protein kinase D family and could bind to diacylglycerol and phorbol lipids, playing critical roles in protein transport, cell migration, proliferation, and epithelial-mesenchymalization [35]. Studies showed that PRKD3 was the major form of PRKD in osteoclasts [36]. Activation of PRKD3 enhanced osteoclast activity, while inhibitors of PRKD3 hindered the differentiation and motility of multinucleated mature osteoclasts [20], and increased trabecular bone volume [36]. Furthermore, PRKD3 promoted cartilage destruction by modulating the expression of FOS/JUN and the production of pro-inflammatory MMP1/13 [19]. The aforementioned evidence all suggested that PRKD3 might be associated with osteoclastogenesis. In our study, we confirmed circPRKD3, the transcription product of PRKD3, was upregulated in response to mechanical stimuli. Interestingly, overexpression of circPRKD3 facilitated early osteogenesis mediated by mechanical force, while downregulation of circPRKD3 exerted the opposite effect. This property which was opposite to the function of its parent gene, might be related to the splicing of exons [37].

So far, it has been discovered that circRNAs can act as sponges of miRNA and RBP, and competitive products during pre-mRNA splicing, mediating gene transcription and protein translation [37]. These different mechanisms depend on the localization of ncRNAs. Studies reported that ncRNAs in the nucleus could directly regulate chromatin, transcription, and alternative splicing. In contrast, ncRNAs in the cytoplasm could act as endogenous competitive RNAs to adsorb miRNAs and RBPs, affecting the stability of mRNAs and indirectly modulating the translation [38]. According to our results, circPRKD3 accounted for a higher cytoplasmic proportion. Incremental evidence has revealed that cytoplastic circRNAs sponge miRNAs to regulate the degradation or translational repression of target mRNAs, especially in the oncology-related process [39, 40]. Therefore, we focused on the ceRNA function of circPRKD3 as a sponge of miRNAs.

miRNAs were highly conserved endogenous ncRNAs that played crucial roles in the regulation of post-transcription, combination of mRNA transcript sequence sites, as well as induction of mRNA degradation and translation [41]. With bioinformatics analysis and multiple software predictions, we identified miR-6783-3p as the potential downstream mechanism of circPRKD3. miR-6783-3p was demonstrated to sponge circ_0006427 and LINC02323 to modulate tumor proliferation, migration, and invasion [42, 43]. In addition, miR-6783-3p could target CYP2C19 to regulate clopidogrel resistance in patients with cardiovascular diseases [44]. However, the mechanobiology and osteogenic role of miR-6783-3p remains unknown. Our research first demonstrated the pro-osteogenic effect of miR-6783-3p under mechanical force. This result was consistent with the function of circPRKD3. Interestingly, when we investigated the mutual regulation between circPRKD3 and miR-6783-3p, we found that only the overexpression of circPRKD3 reduced miR-6783-3p expression, and no significant changes were observed on the contrary. This was possibly due to the fact that circPRKD3 regulated the splicing process of miR-6783-3p and further affected the precursor of miR-6783-3p [45]. However, the above trend was inconsistent with the conclusion reported by Yang et al. that ceRNAs inhibited the expression of each other [46]. Nevertheless, Li et al. showed that although circRNAs and miRNAs could be combined as a sponge, overexpressing either one did not affect the production of the other [47]. Therefore, we thought it was inaccurate to speculate downstream sponge molecules by up-regulating or down-regulating related circRNAs alone. On the one hand, due to the small proportion of intracellular ncRNAs, further knockdown could not show their functions sufficiently. On the other hand, the current testing technology was still limited, and it was unknown whether ncRNAs and miRNAs bound in the sponge state would be cleaved into the free form during sample extraction and affected the development as a result.

It was generally assumed that sponge-bound ncRNAs and miRNAs competed for target proteins and performed opposite biological functions [48, 49]. However, our findings suggested that ncRNAs and miRNAs with similar functions could also act as sponges. Interestingly, Chen et al. also demonstrated that both circ_SPECC1 and miR-526b had anticancer effects, with circ_SPECC1 sponging miR-526b to enhance its inhibition of the KDM4A/YAP1 pathway in gastric cancer cells [50]. Nevertheless, in our study, although circPRKD3 and miR-6783-3p showed a consistent osteogenesis function, the effect of “the whole is greater than the sum of its parts” did not appear when the two factors were co-transfected into PDLSCs. Unexpectedly, the pro-osteogenic effect was attenuated after co-transfection compared with circPRKD3 overexpression alone. This could be due to the complementary base sequences between circPRKD3 and miR-6783-3p preventing the initiation of osteogenesis sites. Additionally, this might also be a homeostasis mechanism to prevent excessive biological activity. Furthermore, although the expression of RUNX2 was inhibited via co-transfection, the prediction of catRAPID and TargetScan displayed no binding sites between the two and RUNX2 sequences. This suggested that the sponge might regulate the osteogenic differentiation of mechanically stimulated PDLSCs in other indirect ways instead of degrading the mRNA of RUNX2.

## Conclusions

Taken together, our study showed that the upregulation of circPRKD3 was associated with osteogenic differentiation of PDLSCs mediated by mechanical force. We also demonstrated that circPRKD3 regulated the osteogenesis process by sponging miR-6783-3p and acting as ceRNAs in mechanically loaded PDLSCs. In addition, we first verified the positive role of miR-6783-3p in bone formation. We believed that circPRKD3, as a vital osteogenesis regulator, could be used to guide healthy and controllable periodontal regeneration and orthodontic tooth movement in the future.

### Electronic supplementary material

Below is the link to the electronic supplementary material.

Additional file 1: Original images of western blots.

Additional file 2: Predicted targets of miR-6783-3p.

Additional file 3: List of abbreviations.

## Data Availability

The datasets used and analyzed during the current study are available from the corresponding author on reasonable request.
