# Supplementary material for: CircPRKD3/miR-6783-3p responds to mechanical force to facilitate the osteogenesis of stretched periodontal ligament stem cells
Source: J Orthop Surg Res. 2024 Apr 22;19:257. doi: 10.1186/s13018-024-04727-7 (PMC11036753; doi:10.1186/s13018-024-04727-7)
Supplement: Supplementary file 1 — Additional file 1: Original images of western blots. [file 13018_2024_4727_MOESM1_ESM.docx]

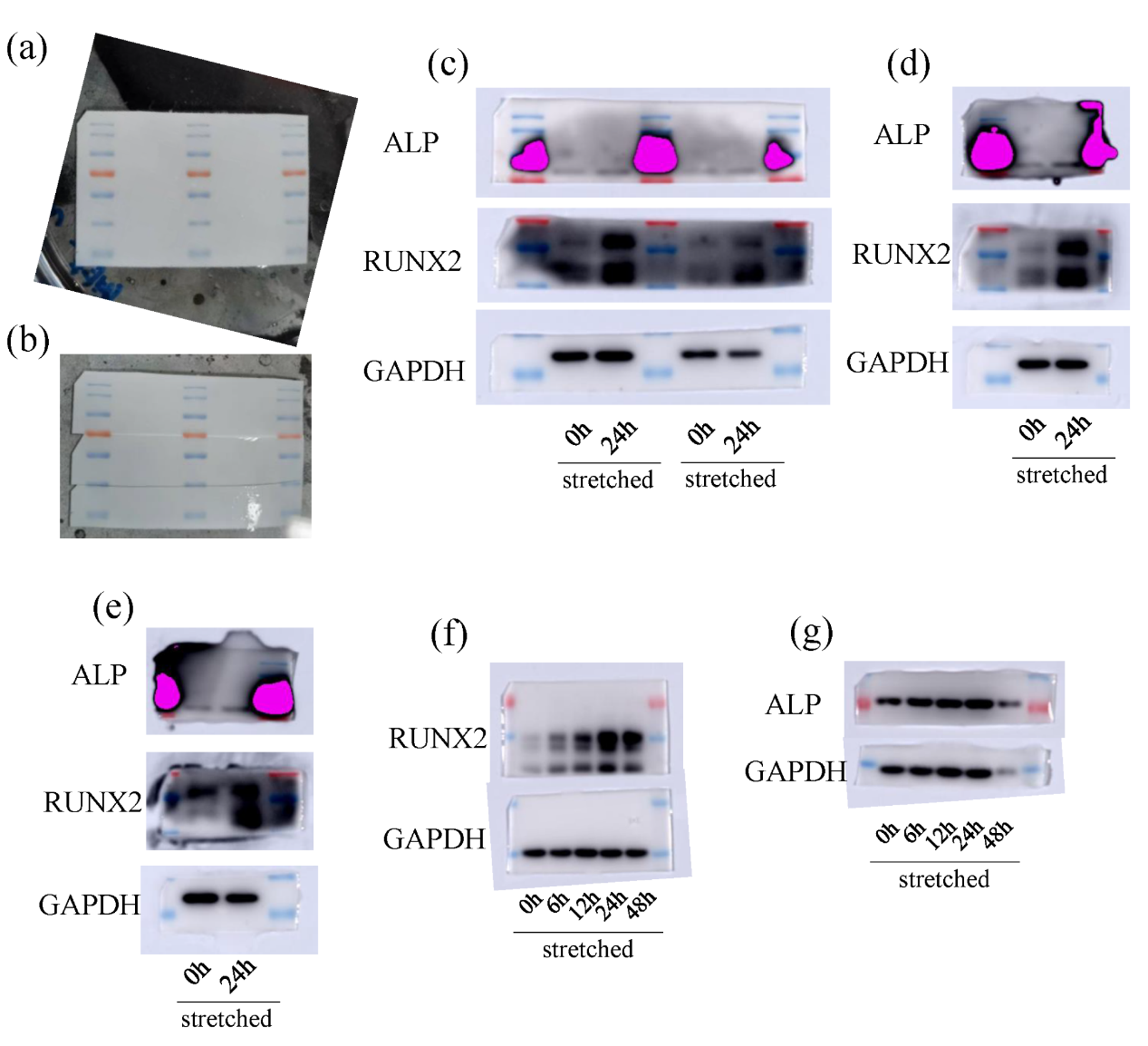


**Supplementary Figure 1. Original images of western blots in Fig. 2G.**

Figure (a) and Figure (b) showed uncropped blot and cropped blot of two different biological samples in Fig. 2G, respectively. Figure (c) showed results of protein bands in Figure (b) with different osteogenic antibodies. When exposing the bands in Figure (c), we found that the band with incubation of ALP antibody was difficult to visualize, so overexposure was performed to show explicit results. To prevent the exposure interference of protein expression difference among different samples, Figure (c) was cropped along the middle marker and was shown as Figure (d) and Figure (e). In the pre-experiment, the osteogenic effect of mechanical force on periodontal ligament stem cells for different time was assayed (Fig. f-g), which also demonstrated the positive role of stretch on osteogenesis. In our original manuscript (Fig. 2G), we use images of protein bands in Figure (d).


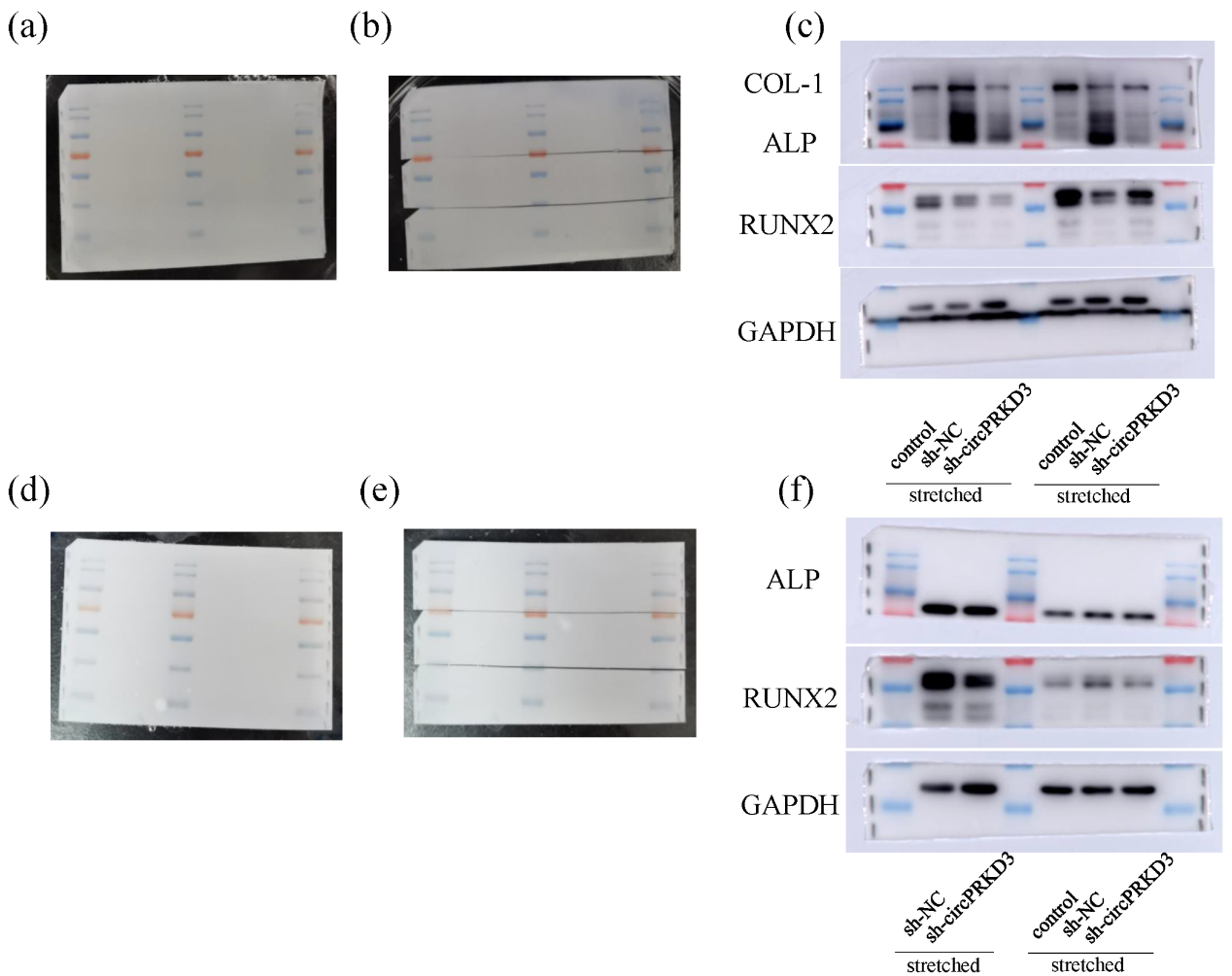


**Supplementary Figure 2. Original images of western blots in** **Fig. 3F.**

Figure (a) and Figure (b) showed uncropped blot and cropped blot of two different biological samples in Fig. 3F, respectively. Figure (c) showed results of protein bands in Figure (b) with different osteogenic antibodies. However, the expression of RUNX2 in the two different samples was opposite. As a result, we used another two different biological samples to verify the osteogenic effect of circPRKD3 knockdown. Figure (d) and Figure (e) showed uncropped blot and cropped blot of another two different biological samples in Fig. 3f, respectively. Figure (f) showed results of protein bands in Figure (e) with different osteogenic antibodies. In our original manuscript (Fig. 3F), we use images of left protein bands in Figure (f).


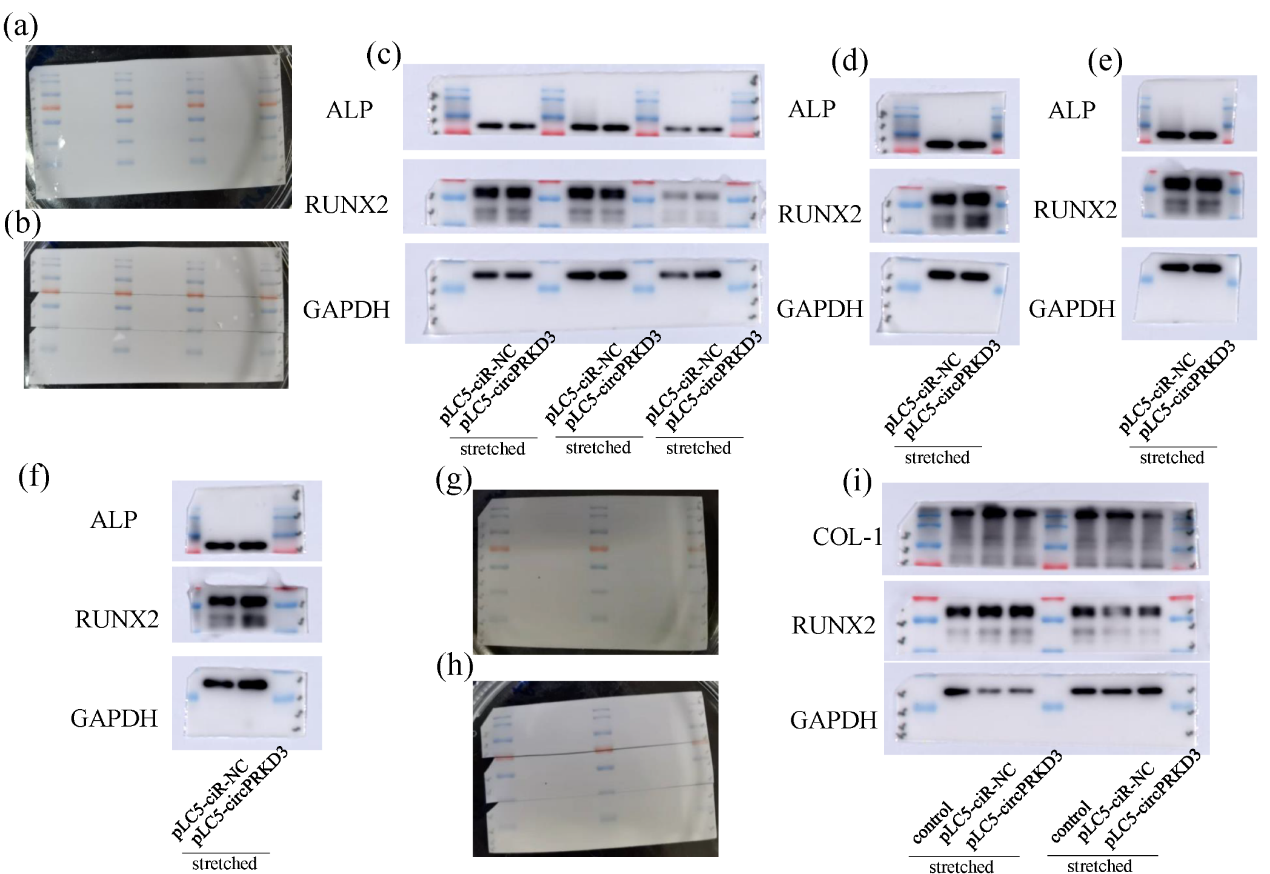


**Supplementary Figure 3. Original images of western blots in Fig. 3P.**

Figure (a) and Figure (b) showed uncropped blot and cropped blot of two different biological samples in Fig. 3P, respectively. Figure (c) showed results of protein bands in Figure (b) with different osteogenic antibodies. To prevent the exposure interference of protein expression difference among different samples, Figure (c) was cropped along the two middle markers and was shown as Figure (d), Figure (e) and Figure (f). However, the RUNX2 expression in the second group was unstable. Therefore, we used another two different biological samples to verify the osteogenic effect of circPRKD3 overexpression. Figure (g) and Figure (h) showed uncropped blot and cropped blot of another two different biological samples in Fig. 3p, respectively. Figure (i) showed results of protein bands in Figure (h) with different osteogenic antibodies. In our original manuscript (Fig. 3P), we use images of protein bands in Figure (d).


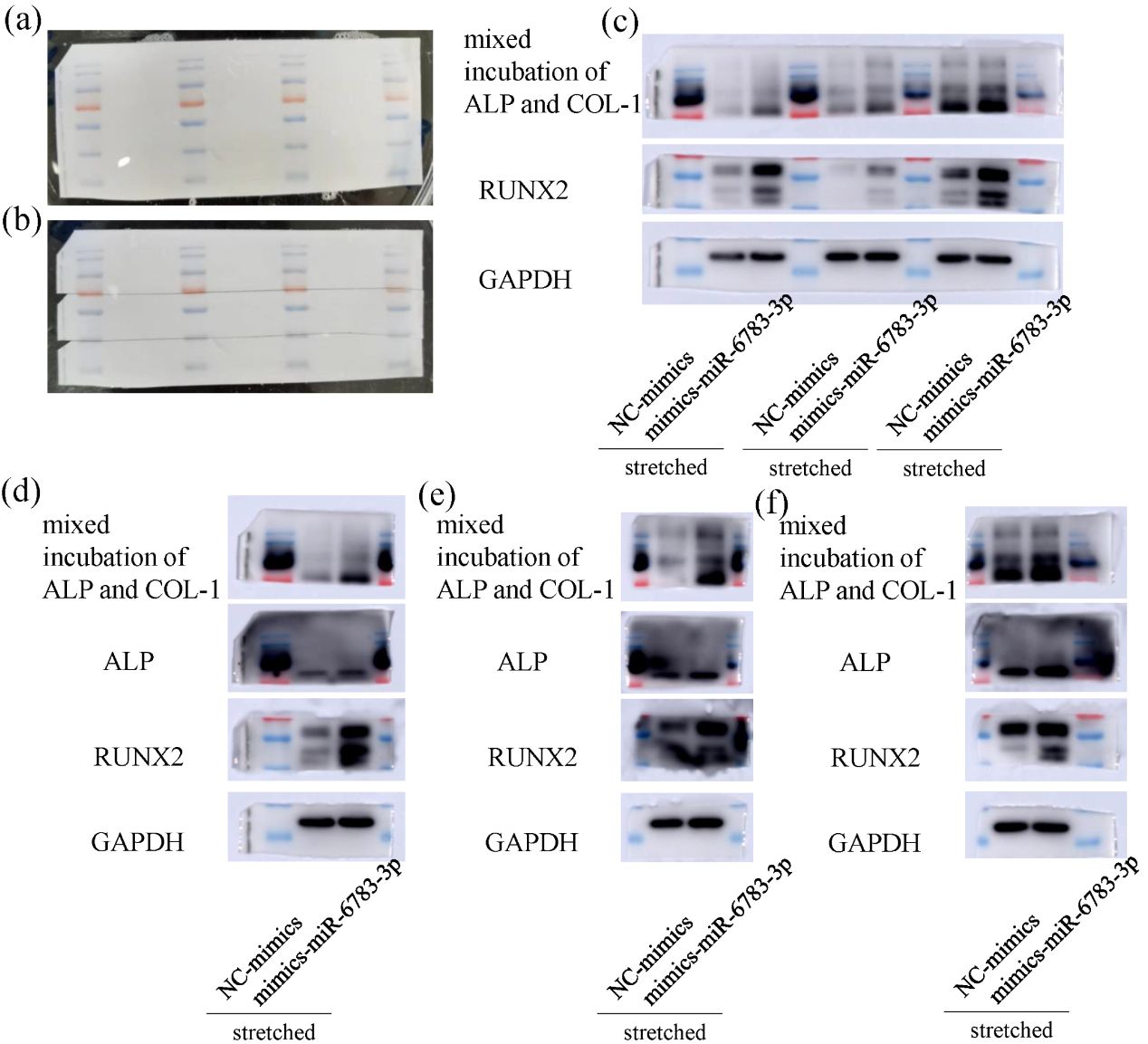


**Supplementary Figure 4. Original images of western blots in** **Fig. 5D.**

Figure (a) and Figure (b) showed uncropped blot and cropped blot of two different biological samples in Fig. 5D, respectively. Figure (c) showed results of protein bands in Figure (b) with different osteogenic antibodies. To prevent the exposure interference of protein expression difference among different samples, Figure (c) was cropped along the two middle markers and was shown as Figure (d), Figure (e) and Figure (f). In our original manuscript (Fig. 5D), we use images of protein bands in Figure (d).


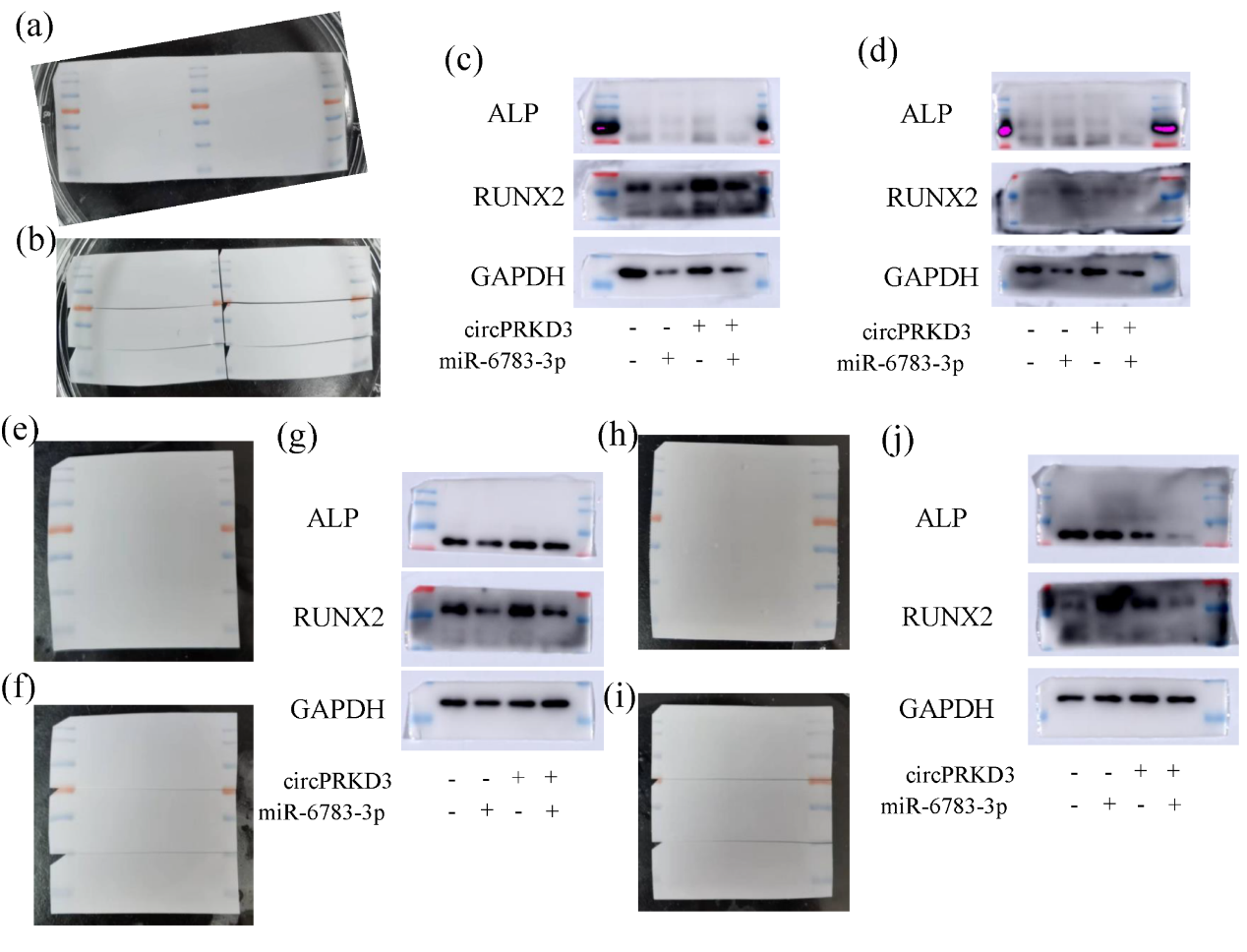


**Supplementary Figure 5. Original images of western blots in** **Fig. 5G.**

Figure (a, e) and Figure (b, f) showed uncropped blot and cropped blot of three different biological samples in Fig. 5G, respectively. Figure (c, d) showed results of protein bands in Figure (b), and Figure (g) showed results of protein bands in Figure (f) with different osteogenic antibodies. However, the expression of ALP and RUNX2 with co-transfection of pLC5‐circPRKD3 plasmids and mimics-miR-6783-3p was unstable in the three samples. Therefore, we used the fourth biological sample to verify the osteogenic effect of co-transfection. Figure (h) and Figure (i) showed uncropped blot and cropped blot of the fourth biological sample in Fig. 5g, respectively. Figure (j) showed results of protein bands in Figure (i) with different osteogenic antibodies. We used results with the same trend in three samples. In our original manuscript (Fig. 5G), we use images of protein bands in Figure (j).
