# Supplementary material for: CircPRKD3/miR-6783-3p responds to mechanical force to facilitate the osteogenesis of stretched periodontal ligament stem cells
Source: J Orthop Surg Res. 2024 Apr 22;19:257. doi: 10.1186/s13018-024-04727-7 (PMC11036753; doi:10.1186/s13018-024-04727-7)
Supplement: Supplementary file 3 — Additional file 3: List of abbreviations. [file 13018_2024_4727_MOESM3_ESM.docx]

**Supplementary Table 2. List of abbreviations**

| abbreviations | interpretation |
| --- | --- |
| circRNAs | circular RNAs |
| circPRKD3 | circular RNA protein kinase D3 |
| PDLSCs | periodontal ligament stem cells |
| qRT‐PCR | quantitative real‐time PCR |
| miRNAs | microRNAs |
| snoRNAs | small nucleolar RNAs |
| rRNAs | ribosomal RNAs |
| tRFs | transfer RNAs |
| lncRNAs | long noncoding RNAs |
| ceRNAs | competing endogenous RNAs |
| MSCs | mesenchymal stem cells |
| ALP | alkaline phosphatase |
| PVDF | polyvinylidene difluoride |
| SD | standard deviation |
| ANOVA | one-way analysis of variance |
